# Effectiveness of Focused Shockwave Therapy versus Radial Shockwave Therapy for Noncalcific Rotator Cuff Tendinopathies: A Randomized Clinical Trial

**DOI:** 10.1155/2021/6687094

**Published:** 2021-01-09

**Authors:** Chengxin Li, Zhizhuo Li, Lijun Shi, Peixu Wang, Fuqiang Gao, Wei Sun

**Affiliations:** ^1^Department of Orthopedics, Peking University China-Japan Friendship School of Clinical Medicine, 2 Yinghuadong Road, Chaoyang District, Beijing 100029, China; ^2^Department of Orthopedics, Graduate School of Peking Union Medical College, China-Japan Friendship Institute of Clinical Medicine, 2 Yinghuadong Road, Chaoyang District, Beijing 100029, China; ^3^Beijing Key Laboratory of Immune Inflammatory Disease, China-Japan Friendship Hospital, 2 Yinghuadong Road, Chaoyang District, Beijing 100029, China

## Abstract

**Background:**

The superiority of focused shockwave therapy (F-SWT) versus radial shockwave therapy (R-SWT) for treating noncalcific rotator cuff tendinopathies remains controversial. This study is aimed at comparing the effectiveness of F-SWT versus R-SWT for the management of noncalcific rotator cuff tendinopathies.

**Methods:**

A total of 46 patients affected by noncalcific rotator cuff tendinopathies were randomly divided into 2 groups of 23 individuals. Patients in group A received 4 sessions of F-SWT, while patients in group B were treated by 4 sessions of R-SWT. In each session, mean energy flux density (EFD) for F-SW 3000 shots was 0.09 ± 0.018 mJ/mm^2^ with 5.1 ± 0.5 Hz, while average pressure for R-SW 3000 shots was 4.0 ± 0.35 bar with 3.2 ± 0.0 Hz. Pain level and shoulder function were assessed with the numerical rating scale (NRS) and Constant-Murley Scale (CMS). The primary endpoint was the change in the mean NRS pain score from baseline to 24 weeks after the intervention. Secondary endpoints were changes in the mean NRS pain scores at all other follow-up points, changes in the mean CMS scores, and radiographic findings.

**Results:**

There were no significant differences between the two groups regarding NRS pain score and CMS score within 24 weeks after intervention (all *p* > 0.05). However, F-SWT resulted in significantly lower NRS compared with R-SWT at 24 weeks and 48 weeks after treatment (2.7 ± 1.0 vs. 4.5 ± 1.2 and 1.4 ± 1.0 vs. 3.0 ± 0.8, respectively, all *p* < 0.001). Similar results were found in CMS changes and radiographic findings.

**Conclusions:**

Both F-SWT and R-SWT are effective in patients with noncalcific rotator cuff tendinopathy. F-SWT proved to be significantly superior to R-SWT at long-term follow-up (more than 24 weeks). This trial is registered with ChiCTR1900022932.

## 1. Introduction

Shoulder pain is frequently encountered in medical practice with the prevalence ranging from 7% to 27% in the general population [1]. Rotator cuff pathology is one of the principal causes of shoulder pain, including calcific and noncalcific tendinopathy [2]. The etiology of noncalcific rotator cuff tendinopathies is still not completely clear but is likely to be the result of a combination of intrinsic and extrinsic factors [3, 4]. Specifically, extrinsic factors cause compression of the rotator cuff tendons and varying degrees of microtraumas, while intrinsic mechanisms are associated with degenerative changes in the rotator cuff tendon. These factors can cause tendon wear and ultimately a part- or full-thickness rotator cuff tear. Patients usually report shoulder pain, being exacerbated by overhead activities and having difficulty reaching behind the back. Diagnosis of this condition is based on the patients' history, physical examination, and radiographic imaging, usually magnetic resonance imaging (MRI) scans, although patients with noncalcific rotator cuff tendinopathies are recommended to attempt conservative treatments such as a course of NSAIDS, physical therapy, and steroid injection before any surgical treatment. Unfortunately, these treatments including surgery hardly prevent tendon degeneration; over half of patients will suffer from recurrent and persistent pain in the long term [5]. Therefore, to compensate for these limitations, alternative therapeutic options for patients with noncalcific rotator cuff tendinopathies are urgently required.

Over the past few decades, extracorporeal shockwave therapy (ESWT) has been widely used for the treatment of musculoskeletal disorders such as nonunion of long-bone fractures, lateral epicondylitis of the elbow, plantar fasciitis, chronic heel pain, and calcific rotator cuff tendinopathies [6]. Multiple mechanisms are involved in the therapeutic effects of ESWT, such as mechanical stimulation, increasing expression of several growth factors, and regional blood flow [7–9]. However, there are two different types of shockwaves in clinic: focused shockwave therapy (F-SWT) and radial shockwave therapy (R-SWT). As described elsewhere [10], focused shockwaves are generated inside the applicator and then focused by a lens and transmitted into the tissue, while radial shockwaves are generated by accelerating a projectile, by means of compressed air, through a tube, at the end of which it hits an applicator that makes contact with the skin. The difference between the technologies in the physical mechanism may lead to the different therapeutic effects between F-SWT and R-SWT. Currently, few studies have directly compared the effects of F-SWT versus R-SWT on noncalcific rotator cuff tendinopathies. Therefore, This study is aimed at comparing the effectiveness of two different types of shockwaves and determining the superior therapeutic method for noncalcific rotator cuff tendinopathies.

## 2. Methods

### 2.1. Ethical Approval

This prospective randomized controlled trial was approved by the Ethics Committee of China-Japan Friendship Hospital (No. 2009-2012), and it was registered at the Chinese Clinical Trial Registry (No. ChiCTR1900022932). Our study was conducted in accordance with the Declaration of Helsinki. Written informed consent was obtained from each participant.

### 2.2. Study Design and Population

From June 2018 to February 2020, subjects who were diagnosed with noncalcific rotator cuff tendinopathies in the outpatient department of our hospital were asked to participate in the study. Subjects were regarded eligible if they met the following inclusion criteria: (1) between 21 and 75 years old; (2) baseline NRS pain score of 5 or higher; (3) pain in the shoulder and/or exacerbation of pain with overhead-throwing activity; (4) decreased range of motion with shoulder flexion, abduction, and internal and external rotation; (5) these symptoms had to be present for at least 3 months; and (6) MRI found only intensity changes in the rotator cuff and absence of full-thickness tears in the tendon. The diagnosis was primarily based on history and MRI; however, X-ray was used to exclude calcific rotator cuff tendinopathies. Patients were excluded if they met any of the following criteria: (1) significant atrophy of any of the shoulder girdle muscles; (2) failed previous focused or radial ESWT in the affected shoulder; (3) previous surgery in the affected shoulder; (4) recent (<6 weeks) corticosteroid injection, needling, or nerve block in the shoulder region; (5) recent (<3 months) platelet-rich plasma or stem cell injection in the affected shoulder; (6) current cancer, or cancer in the past 5 years; (7) coagulation abnormalities, or current prescription strength anticoagulation medication (prophylactic daily dose of acetylsalicylic acid is allowed); and (8) presence of any condition or abnormality that in the opinion of the investigator would compromise the safety of the patient or the quality of the data.

Based on previously reported studies [11], we assumed a standard deviation of 2 points of NRS in the F-SWT group and 1.5 points in the R-SWT group at 24 weeks. A difference in the NRS pain score of 1.5 points at 24 weeks was considered clinically relevant. With a power of 80% and two-sided alpha of 5%, 21 shoulders per group were needed to detect a clinically relevant difference.

Subjects were randomly allocated to either F-SWT or R-SWT groups based on a computer-generated randomized block design. Block randomization was utilized to balance the group with a block size of 4 and F-SWT: R-SWT ratio of 1 : 1 in each block. The randomization allocation was sealed in opaque envelopes, and the clinical coordinator did not know which group the subject would be assigned to until the envelopes were opened. The assessors and statisticians were blinded until the completion of the study. It is impossible to blind the patients and therapist because the machines can make different sounds.

### 2.3. Extracorporeal Shockwave Therapy Procedure

The regions of tendinopathy were identified by MRI, and the corresponding surfaces of the regions were marked. The patients were asked to sit and took some special activities to expose the lesions: with his hands behind his back (arm bent and internally rotated), to expose the supraspinatus tendon; hands in front with the arm externally rotated to expose the subscapularis tendon; and hands in front with the arm slightly internally rotated to expose infraspinatus tendon and teres minor tendon.

Each patient will receive ESWT (F-SWT or R-SWT) once a week (5-9 days interval between each session), for a total of 4 sessions. Every session consists of 3000 shockwaves, targeted to the marked area. F-SWT will be performed using the Dornier Aries device which was an electromagnetic device at level 2-10 (energy flux density EFD = 0.01‐0.15 mJ/mm^2^). We started at level 2 within the beginning of 160 shots. Then, we raised one energy level every 160 shots up to a maximum of level 10. All remaining shocks are at the maximum energy level. In general, the maximum energy that people can tolerate is between level 6 and level 10 (level 2 with EFD = 0.013 mJ/mm^2^ and frequency = 16 Hz; level 3 with EFD = 0.028 mJ/mm^2^ and frequency = 10 Hz; level 4 with EFD = 0.051 mJ/mm^2^ and frequency = 8 Hz; level 5 with EFD = 0.062 mJ/mm^2^ and frequency = 6 Hz; level 6 with EFD = 0.084 mJ/mm^2^ and frequency = 5 Hz; level 7 with EFD = 0.096 mJ/mm^2^ and frequency = 5 Hz; level 8 with EFD = 0.117 mJ/mm^2^ and frequency = 4 Hz; level 9 with EFD = 0.130 mJ/mm^2^ and frequency = 4 Hz; level 10 with EFD = 0.150 mJ/mm^2^ and frequency = 4 Hz). As a result, the mean energy flux density for the 3000 focused shots was 0.09 ± 0.018 (mean ± SD) mJ/mm^2^, and the frequency is 5.11 ± 0.46 Hz (mean ± SD).

R-SWT will be performed using the ZhuHai Hema device with an R15 applicator whose diameter was 15 mm at 1-5 bar. Each session consists of 3000 shots. We started at 1.0 bar within the beginning of 200 shots. Then, we raised one energy level every 200 shots up to a maximum of level 5.0 bar. In general, the maximum energy that people can tolerate is between level 4.0 and 5 bar (level 1.0 bar frequency = 5 Hz; level 2.0 bar frequency = 5 Hz; level 3.0 bar frequency = 4 Hz; level 4.0 bar frequency = 3 Hz; level 5.0 bar frequency = 3 Hz). The mean pressure was 4.0 ± 0.35 bar (mean ± SD), and the frequency is 3.2 ± 0.0 Hz (mean ± SD). Ultrasound coupling gel was used to minimize the loss of shockwave energy at the interface between the applicator head and the skin. Start at a low energy (i.e., F-SWT level 2; R-SWT 1.0 bar) and increase the energy level up to the maximum tolerable within 500 shockwaves. Then, increase the energy level slowly, up to a maximum of level 10 (F-SWT device), 5 bar (R-SWT device). No local anesthesia, analgesics, or nonsteroidal anti-inflammatory drugs were used during the procedure. Because the immediate inflammatory action of ESWT may be important for efficacy in musculoskeletal pain conditions, NSAID use is strongly discouraged during the 4-week treatment period. After the intervention, patients in both groups were required to active their shoulders immediately. They can do daily activities but avoid lifting heavy objects until 12 months later. No other therapies were allowed until they finished the study.

### 2.4. Posttreatment Follow-Up and Outcome Measures

All subjects will receive follow-up visits at 4, 12, 24, and 48 weeks after the final treatment session. The primary endpoint was the change of the numerical rating scale (NRS) of pain from baseline to week 24. The 11-point NRS (0 = no pain, 10 = maximum pain) has been recommended as a primary endpoint for chronic pain studies by the Initiative on Methods, Measurement, and Pain Assessment in Clinical Trials [12]. Secondary end points were changes of NRS of pain at all other follow-up points and changes of the mean Constant and Murley Scale (CMS) score at 4, 12, 24, and 48 weeks. The CMS (score of 0 to 100; lower score indicates poorer function) is a standardized simple clinical method of assessing shoulder function. It has been extensively validated and shows good intra- and interobserver reproducibility [13]. The CMS combines subjective and objective measurements in one score. Subscales of CMS include pain, activities of daily living (ADL), range of motion (ROM), and power. The CMS score increases as shoulder mobility increases and pain decreases; therefore, the higher the CMS, the greater the improvement in the quality of life of the patient.

**Figure 1:**
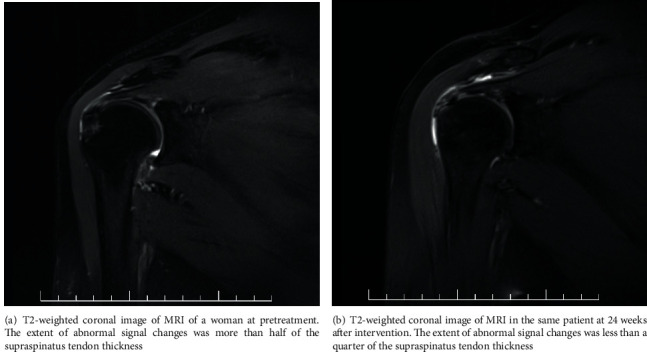
MRI findings of supraspinatus tendinopathy.

The MRI examinations were performed based on a standardized protocol for the evaluation of rotator cuff pathology. All patients were placed in the supine position with the arm in external rotation. Proton density, T1-weighted, T2-weighted, and fat-saturated spin-echo images were obtained in oblique, coronal, axial, and sagittal imaging planes. This was achieved by using a 16-channel phased-array sensitivity-encoding (SENSE) body coil on a Philips Ingenia 3.0-T MRI machine (Philips Medical Systems, Best, Netherlands). MRI images will be obtained at pretreatment, 24 and 48 weeks after intervention. Images were evaluated by one experienced musculoskeletal radiologist; any ambiguity was resolved through discussion with another radiologist, and two radiologists had to come to a consensus. All the radiologists were blinded to the groups in the process. Rotator cuff tendinopathies were graded according to the criteria established by Sein et al. [14, 15]. In the largest plains of tendinopathy, abnormal signal intensities on T2-weighted images were classified from grades I to IV according to the extent of the signal changes. Grade I was a normal tendon, without abnormal signal intensity. In grade II, the abnormal signal intensity was less than 25% of the tendon thickness; in grade III, less than 50%; and in grade IV, more than 50% (Figure 1).

### 2.5. Statistical Analysis

All data analyses were performed using SPSS version 20.0 software (SPSS; Chicago, IL, USA). Continuous variables were presented as means ± standard deviations, and categorical variables were presented as counts. Comparisons between groups were performed using the unpaired *t*-test, Mann-Whitney *U*-test, chi-square test, and Fisher's exact test, as appropriate. Computed *p* values were two-sided, and *p* < 0.05 was considered statistically significant.

## 3. Results

### 3.1. Patient Demographics

**Figure 2:**
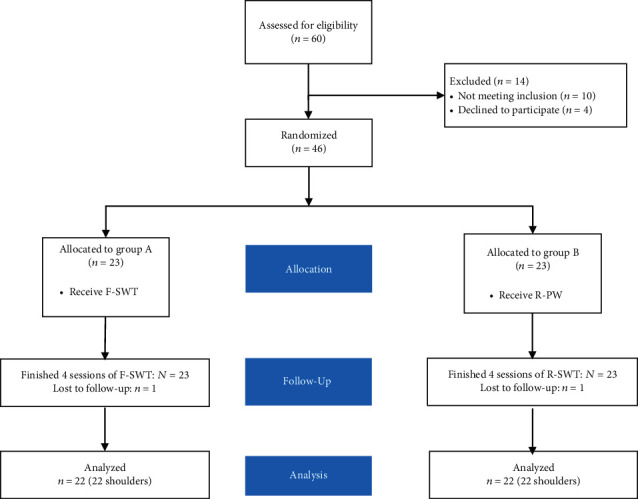
The flow chart of participants through this trail.

From June 2018 to February 2019, 60 patients with noncalcific rotator cuff tendinopathies were assessed for eligibility. Ten patients were excluded according to exclusion criteria; four patients were reluctant to join in the trial, leaving 46 patients for the study. After randomization, there were 23 patients in each group. All patients finished the treatment protocol. After the 48-week follow-up period, each group lost one patient because of changed contact information, so the total of 44 patients entered the result analysis finally (Figure 2). The baseline characteristics of the study population are presented in Table 1. Demographic and baseline characteristics did not differ significantly (all *p* > 0.05) between the two randomized groups.

### 3.2. Primary Outcome Measure

At 24 weeks after intervention, the mean NRS pain score decreased from 5.9 ± 0.9 to 2.7 ± 1.0 in group A, while from 5.5 ± 0.7 to 4.5 ± 1.2 in group B. Moreover, the difference between the two groups is statistically significant (*p* < 0.001; Table 2).

### 3.3. Secondary Outcome Measure

There were no significant differences of NRS pain scores between the two groups at 4 weeks and 12 weeks after treatment (all *p* > 0.05). Another significant difference of NRS pain scores was seen at 48 weeks between the F-SWT group and the R-SWT group (1.4 ± 1.0 vs. 3.0 ± 0.8, *p* < 0.001, Table 2).

Table 3 presents the changes of CMS scores of the two groups over time. These results parallel our presentation of the NRS results. Significant differences of total CMS scores between F-SWT and R-SWT were only observed at 24 weeks (79.6 ± 5.6 vs. 75.0 ± 5.4, *p* = 0.007) and 48 weeks (83.6 ± 6.0 vs. 76.8 ± 6.5, *p* = 0.001). The subscales such as pain, ADL, and ROM are generally consistent with the total CMS score. At 24 weeks, significantly higher scores for pain and ADL were observed in the F-SWT group. At 48 weeks, all subscales (except power) showed significantly higher scores in the F-SWT group.

### 3.4. MRI Findings

Grades of MRI findings in tendinopathy are presented in Table 4. MRI findings showed grade II or III in 91% (20/22) of group A while 95% (21/22) of group B at baseline. At 24 weeks after intervention, 82% (18/22) of patients in group A and 23% (5/22) of patients in group B experienced at least one grade of decreasing of MRI findings. Consequently, there are 4 patients with grade I, 12 patients with grade II, and 6 patients with grade III in group A, while 2 patients with grade I, 6 patients with grade II, and 14 patients with grade III in group B (*p* = 0.024). After 48 weeks, 100% (22/22) of patients in group A and 50% (11/22) of patients in group B decreased more than one grade of MRI findings in tendon. As a result, 100% (22/22) of patients in group A are with grade I or II while 82% (18/22) of patients in group B are with grade II or III (*p* = 0.032).

### 3.5. Adverse Events

An adverse event (AE) is defined as an untoward medical occurrence in a clinical investigation, and it does not necessarily imply a causal relationship with the treatment. Adverse effects were assessed by clinical examination, MRI scan, and patients' feedback. Patients were explicitly asked to report any skin reddening, bruising, ecchymosis, small hematoma, welling, paresthesia, hypoesthesia at the treatment site, myalgia, or rash or itching caused by reaction to ultrasound gel. Patients were also asked to report any other adverse events. In the F-SWT group, 5 patients reported moderate pain, 1 patient reported syncope, and 2 patients occurred skin redness. In the R-SWT group, 3 patients reported moderate pain and 1 reported migraine. No other adverse effects were noted in both groups.

## 4. Discussion

Recently, surgery of rotator cuff tendinopathy is gradually being superseded by new options focusing on minimally invasive [16]. Several studies have proved the beneficial effect of ESWT for calcific tendinopathy of the rotator cuff [17–19]. However, there is still controversy regarding the use of this technique in noncalcific tendinopathy. A recent systematic review of the literature on the effectiveness of ESWT identified studies that had good treatment results [20]. Based on these studies, it was concluded that ESWT seems to be a safe and promising treatment for noncalcific rotator cuff tendinopathies. Therefore, the first aim of this present study was not to answer the question whether ESWT is effective for this condition, but to compare the effectiveness of two different ESWTS.

According to our study, both types of ESWT can relieve the pain immediately after therapy. The beneficial effect appeared significantly earlier than those in the traditional conservative therapy such as nonsteroidal anti-inflammatory drugs and local corticosteroid injections [21]. There are several theories that can partly explain the immediate pain relief by ESWT. One is that shockwaves stimulate the nociceptors to fire high-frequent nerve impulses. Propagation of nerve impulses is blocked according to the gate-control theory [22]. Therefore, we speculate that the effect of high-frequency shockwave is better than that of low frequency. However, our results suggested that the effect of F-SW with roughly 5 Hz is similar to R-SW with roughly 3 Hz in short term, which indicates the difference of frequency in our study is not enough to make a difference in effect. Shockwaves can also distort parts of the total cell membrane. The nociceptors cannot build up a generator potential; thus, pain sensation is avoided [23]. Some researchers believe that shockwaves can change the chemical environment of the cell membrane by generating free radicals, which in turn result in pain-inhibiting chemicals near the cells. Maier et al. [24] proved a decreased concentration of substance P and prostaglandin E2 in the periosteum covering the cortical femur surface after high-energy extracorporeal shockwave application to the distal femur in the rabbit.

However, there are no statistically significant differences between the two groups until 24 weeks after the intervention. The results are similar to previous studies exploring the effectiveness of the two types of ESWT on other diseases. The trial by van der Worp et al. [25] compared the effectiveness of F-SWT and R-SWT for treating patellar tendinopathies. After 14 weeks of follow-up, the results showed that no significant differences in effectiveness were observed between the two groups. Similar results can also be found in the trial of Król [26]. After 12 weeks of follow-up, both focused and radial shockwave therapies can comparably and gradually reduce pain in subjects with tennis elbow with no significant differences. Notably, there are some defects that may have limited the generalizability of their studies. First, the follow-up is too short to find the differences. Second, none of them have any imaging evidence to support that the lesion has healed or will not continue to change at the end of the trail.

The most important finding of the present study was that focused shockwaves appeared to be significantly superior to radial shockwaves at 24 weeks and 48 weeks. Several studies [27–29] also indicated that the beneficial effect of F-SWT would exist until the 12-month follow-up. Since this is the first study to directly compare the effect of the two types of ESWT on noncalcific shoulder tendinopathy and followed up to 48 weeks, the reasons why focused shockwave is significantly superior to radial shockwaves at long-time follow-up are still uncertain. Indeed, it has been proved that the biological effects are related to the pressure wave from [30]. BrañEs et al. [31] proved that F-SWT was associated with increased neovascularization in rotator cuff tendinopathies. Tei et al. [32] showed that CD34+ mononuclear cells were able to incorporate into neoangiogenic foci and participated in ligament tissue repair. Besides, angiogenesis can minimize extrinsic scarring and improve muscle and tendon-to-bone healing, thus improving the tendon attachment strength [33]. These slow processes can partially explain the function improvement at 24 weeks and 48 weeks not solely because of pain relief. However, the radial shockwave is a low- to medium-energy shockwave that transmits radially from the tip of the applicator to the target zone. The pressure and energy density decrease during penetrating in the tissue. As a result, the modes of action and the effects of R-SWT on living tissue may differ from those of F-SWT. Many authors [34] reported that histological reaction to the ESWT depended on the total energy delivered to the tissue (total effectiveness energy = EFD [mJ/mm^2^] × mm^2^ × number) (mJ). However, considering the patient's tolerance to ESWT, R-SWT with medium EFD (4.0 ± 0.35 bar) and F-SWT with low EFD (0.09 ± 0.018 mJ/mm^2^) were used in our study. Using different energy levels may cause unequal total energy delivered to the tissue. According to previous studies [35], R-SW with 4.0 bar may be insufficient since the thickness of tissue in the shoulder can prevent the applicator from reaching an effective depth. These possible reasons can partly explain our results that low energetic F-SWT seemed more effective than medium energetic R-SWT. If the same energy was used in the F-SWT and R-SWT, the superior therapeutic effect of F-SWT may be more obvious.

In addition, our results also showed that all subscales (except power) of CMS are generally in line with the total CMS scores. According to Stiller and Uhl [36], the subscale of power ignores the individual differences caused by gender and age. Patel et al. [37] suggested to remove the subscale of the muscle strength and adjust it to a score of 75 points, known as the adjusted CMS, which was recognized to avoid a large difference in scores among different groups. Therefore, although the subscales of power in F-SWT were not better than in R-SWT, we still think of a greater improvement of the condition and quality of life in the R-SWT group.

We also found patients treated by F-SWT showed more radiological improvement than R-SWT. The reduction of high signal intensity in T2 paralleled to pain relief and function improvement. This makes the results more reliable. Miniaci and Salonen believed that MRI was the golden standard for the diagnosis of noncalcific shoulder tendinopathies [38]. Kjellin et al. [39] showed that degenerative changes in the rotator cuff of the cadaveric shoulder are related to increased signal intensity at MRI imaging. Seo et al. [40] also used MRI to document long-term outcomes of extracorporeal shockwave therapy on gluteal tendinopathy, and the results proved it is reliable.

At last, this study provides some suggestions for the use of ESWT in clinical practice. The introduction of R-SWT and F-SWT made ESWT more affordable and easier to administer. However, there is no agreement in the literature as to which ESWT is more effective for tendinopathies. Based on our study, we recommend F-SWT to treat noncalcific rotator cuff tendinopathies. We had better to try this therapy before more radical options like surgery.

This study is a prospective randomized study with a rigorous design, a clinically feasible intervention, and sufficient follow-up. However, there are still some limitations as follows. First, we roughly compared low energetic (0.09 mJ/mm^2^) focused ESWT with 5 Hz to medium energetic (4.0 bar approximately correspond to 0.2 mJ/mm^2^) radial ESWT with 3 Hz. The effect of different energy levels has been considered and discussed in the study; however, different frequencies may also lead to the unequal effect. Further studies are needed to explore the relationship between therapeutic effect and frequency of ESWT. Second, our study is absent of one placebo group receiving sham shockwave therapy. However, it is difficult to create a placebo group, as people generally know that shockwave therapy elicits strong physical sensations and it is an unethical practice to use sham therapy on patients. Third, we lack a control group to test the results of other conservative therapies such as nonsteroidal anti-inflammatory drugs and local corticosteroid injections. Thus, we cannot learn whether the two types of shockwaves will provide more benefits than the other conservative therapies. Last but not the least, we performed the study in a small number of patients, so statistical analyses could be affected by a low sample size.

## 5. Conclusion

Low energetic F-SWT appeared to be more effective than medium energetic R-SWT at long-term follow-up (more than 24 weeks). Based on the present clinical results, we recommend F-SWT to treat noncalcific rotator cuff tendinopathies. Our findings need to be confirmed in high-quality randomized controlled trials.

## Figures and Tables

**Table 1:** Baseline demographics and clinical characteristics.

Characteristics	Group A (*n* = 22)	Group B (*n* = 22)	*p* value
Gender (male/female)	10/12	9/13	0.76^‡^
^∗^Age (years)	50.6 ± 5.2	53.4 ± 6.7	0.45^†^
Affected side (right/left)	18/4	20/2	0.66^‡^
^∗^Duration of symptoms (weeks)	14.3 ± 2.2	14.0 ± 2.4	0.78^†^
^∗^CMS score (100 points)	66.7 ± 6.2	65.8 ± 4.7	0.57^†^
^∗^Pain (15 points)	7.7 ± 2.5	8.2 ± 2.5	0.55^†^
^∗^ADL (20 points)	14.2 ± 2.2	13.5 ± 2.3	0.32^†^
^∗^ROM (40 points)	30.3 ± 3.4	30.1 ± 3.2	0.86^†^
^∗^Power (25 points)	14.5 ± 4.0	14.0 ± 4.3	0.67^†^
^∗^NRS pain score (10 points)	5.9 ± 0.9	5.5 ± 0.7	0.20^†^

^∗^The values are given as mean ± standard deviation; ^†^unpaired *t*-test; ^‡^Monte Carlo or Fisher exact test. CMS: Constant and Murley Scale; ADL: activity of daily living; ROM: range of motion; NRS: numerical rating scale.

**Table 2:** Changes of the NRS pain score from the baseline to 48 weeks after intervention.

Measure	Time	Group A (F-SWT)	Group B (R-SWT)	*p* value^†^
NRS	Baseline	5.9 ± 0.9	5.5 ± 0.7	*p* = 0.20
	4-week	4.8 ± 0.8	4.8 ± 0.9	*p* = 0.73
	12-week	3.9 ± 1.4	4.5 ± 1.0	*p* = 0.07
	24-week	2.7 ± 1.0	4.5 ± 1.2	*p* < 0.001
	48-week	1.4 ± 1.0	3.0 ± 0.8	*p* < 0.001

NRS: numerical rating scale; F-SWT: focused shockwave therapy; R-SWT: radial shockwave therapy. ^†^Unpaired *t*-test.

**Table 3:** Changes of the CMS score and its components from the baseline to 48 weeks after intervention.

Measure	Time	Group A (F-SWT)	Group B (R-SWT)	*p* value^†^
Total CMS score	Baseline	66.7 ± 6.2	65.8 ± 4.7	*p* > 0.05
	4-week	70.5 ± 4.3	70.3 ± 4.7	*p* > 0.05
	12-week	73.6 ± 6.0	71.2 ± 5.2	*p* > 0.05
	24-week	79.6 ± 5.6	75.0 ± 5.4	*p* = 0.007
	48-week	83.6 ± 6.0	76.8 ± 6.5	*p* = 0.001

Pain	Baseline	7.7 ± 2.5	8.2 ± 2.5	*p* > 0.05
	4-week	9.1 ± 2.5	9.3 ± 2.8	*p* > 0.05
	12-week	10.2 ± 2.4	10.0 ± 2.7	*p* > 0.05
	24-week	12.7 ± 2.5	10.7 ± 2.8	*p* = 0.015
	48-week	13.6 ± 2.3	11.6 ± 2.8	*p* = 0.012

ADL	Baseline	14.2 ± 2.2	13.5 ± 2.3	*p* > 0.05
	4-week	15.0 ± 1.8	14.1 ± 2.5	*p* > 0.05
	12-week	15.4 ± 2.1	14.4 ± 2.5	*p* > 0.05
	24-week	17.0 ± 2.0	14.7 ± 2.6	*p* = 0.002
	48-week	17.7 ± 1.9	16.0 ± 2.4	*p* = 0.016

ROM	Baseline	30.3 ± 3.4	30.1 ± 3.2	*p* > 0.05
	4-week	31.4 ± 3.0	32.5 ± 2.6	*p* > 0.05
	12-week	32.1 ± 3.1	32.8 ± 2.4	*p* > 0.05
	24-week	34.3 ± 2.2	33.9 ± 2.9	*p* > 0.05
	48-week	36.0 ± 3.0	34.1 ± 2.9	*p* = 0.035

Power	Baseline	14.5 ± 4.0	14.0 ± 4.3	*p* > 0.05
	4-week	15.1 ± 3.3	14.6 ± 3.5	*p* > 0.05
	12-week	15.9 ± 2.9	14.8 ± 3.8	*p* > 0.05
	24-week	15.9 ± 2.6	15.7 ± 3.3	*p* > 0.05
	48-week	16.3 ± 2.6	15.1 ± 3.1	*p* > 0.05

CMS: Constant and Murley Scale; ADL: activity of daily living; ROM: range of motion; F-SWT: focused shockwave therapy; R-SWT: radial shockwave therapy. ^†^Unpaired *t*-test.

**Table 4:** Grade of MRI findings of rotator cuff tendon.

Time	Grade of MRI in group A (*n* = 22)				Grade of MRI in group B (*n* = 22)				*p* value^†^
	I	II	III	IV	I	II	III	IV	
Baseline	0	4	16	2	0	6	15	1	*p* = 0.394
6 months	4	12	6	0	2	6	14	0	*p* = 0.024
12 months	8	14	0	0	4	13	5	0	*p* = 0.032

MRI: magnetic resonance imaging. ^†^ Mann-Whitney U-test.

## Data Availability

The datasets used and/or analyzed during the present study are available from the corresponding author on reasonable request.

## References

[1] Ackerman I. N., Page R. S., Fotis K. (2018). Exploring the personal burden of shoulder pain among younger people in Australia: protocol for a multicentre cohort study. *BMJ open*.

[2] Lewis J. S. (2009). Rotator cuff tendinopathy/subacromial impingement syndrome: is it time for a new method of assessment?. *British Journal of Sports Medicine*.

[3] Seitz A. L., McClure P. W., Finucane S., Boardman N. D., Michener L. A. (2011). Mechanisms of rotator cuff tendinopathy: Intrinsic, extrinsic, or both?. *Clinical Biomechanics*.

[4] Federer A. E., Steele J. R., Dekker T. J., Liles J. L., Adams S. B. (2017). Tendonitis and Tendinopathy: What Are They and How Do They Evolve?. *Foot and Ankle Clinics*.

[5] Winters J. (1999). The long-term course of shoulder complaints: a prospective study in general practice. *Rheumatology*.

[6] Schmitz C., Császár N. B. M., Milz S. (2015). Efficacy and safety of extracorporeal shock wave therapy for orthopedic conditions: a systematic review on studies listed in the PEDro database. *British Medical Bulletin*.

[7] Link K. A., Koenig J. B., Silveira A., Plattner B. L., Lillie B. N. (2013). Effect of unfocused extracorporeal shock wave therapy on growth factor gene expression in wounds and intact skin of horses. *American Journal of Veterinary Research*.

[8] Kuo Y.-R., Wang C.-T., Wang F.-S., Chiang Y.-C., Wang C.-J. (2009). Extracorporeal shock-wave therapy enhanced wound healing via increasing topical blood perfusion and tissue regeneration in a rat model of STZ-induced diabetes. *Wound Repair and Regeneration*.

[9] Rosso F., Bonasia D. E., Marmotti A., Cottino U., Rossi R. (2015). Mechanical Stimulation (Pulsed Electromagnetic Fields “PEMF” and Extracorporeal Shock Wave Therapy “ESWT”) and Tendon Regeneration: A Possible Alternative. *Frontiers in Aging Neuroscience*.

[10] Ogden J. A., Tóth-Kischkat A., Schultheiss R. (2001). Principles of Shock Wave Therapy. *Clinical Orthopaedics and Related Research*.

[11] Bannuru R. R., Flavin N. E., Vaysbrot E., Harvey W., McAlindon T. (2014). High-Energy Extracorporeal Shock-Wave Therapy for Treating Chronic Calcific Tendinitis of the Shoulder. *Annals of Internal Medicine*.

[12] Dworkin R. H., Turk D. C., Farrar J. T. (2005). Core outcome measures for chronic pain clinical trials: IMMPACT recommendations. *Pain*.

[13] CONSTANT C. R. (1997). AN EVALUATION OF THE CONSTANT-MURLEY SHOULDER ASSESSMENT. *The Journal of Bone and Joint Surgery. British volume*.

[14] Sein M. L., Walton J., Linklater J. (2007). Reliability of MRI assessment of supraspinatus tendinopathy. *British Journal of Sports Medicine*.

[15] Vasishta A., Kelkar A., Joshi P., Hapse R. (2019). The value of sonoelastography in the diagnosis of supraspinatus tendinopathy—a comparison study. *The British Journal of Radiology*.

[16] Louwerens J. K. G., Veltman E. S., van Noort A., van den Bekerom M. P. J. (2016). The Effectiveness of High-Energy Extracorporeal Shockwave Therapy Versus Ultrasound-Guided Needling Versus Arthroscopic Surgery in the Management of Chronic Calcific Rotator Cuff Tendinopathy: A Systematic Review. *Arthroscopy: The Journal of Arthroscopic & Related Surgery*.

[17] Peters J., Luboldt W., Schwarz W., Jacobi V., Herzog C., Vogl T. J. (2004). Extracorporeal shock wave therapy in calcific tendinitis of the shoulder. *Skeletal Radiology*.

[18] Daecke W., Kusnierczak D., Loew M. (2002). Long-term effects of extracorporeal shockwave therapy in chronic calcific tendinitis of the shoulder. *Journal of shoulder and elbow surgery*.

[19] Farr S., Sevelda F., Mader P., Graf A., Petje G., Sabeti-Aschraf M. (2011). Extracorporeal shockwave therapy in calcifying tendinitis of the shoulder. *Knee Surgery, Sports Traumatology, Arthroscopy*.

[20] Huisstede B. M. A., Gebremariam L., van der Sande R., Hay E. M., Koes B. W. (2011). Evidence for effectiveness of Extracorporal Shock-Wave Therapy (ESWT) to treat calcific and non-calcific rotator cuff tendinosis - A systematic review. *Manual Therapy*.

[21] Skedros J. G., Hunt K. J., Pitts T. C. (2007). Variations in corticosteroid/anesthetic injections for painful shoulder conditions: comparisons among orthopaedic surgeons, rheumatologists, and physical medicine and primary-care physicians. *BMC Musculoskeletal Disorders*.

[22] Melzack R., Wall P. D. (1996). Pain mechanisms: A new theory: A gate control system modulates sensory input from the skin before it evokes pain perception and response. *Pain Forum*.

[23] Wess O. J. (2008). A neural model for chronic pain and pain relief by extracorporeal shock wave treatment. *Urological research*.

[24] Maier M., Averbeck B., Milz S., Refior H. J., Schmitz C. (2003). Substance P and prostaglandin E2 release after shock wave application to the rabbit femur. *Clin Orthop Relat Res*.

[25] van der Worp H., Zwerver J., Hamstra M., van den Akker-Scheek I., Diercks R. L. (2014). No difference in effectiveness between focused and radial shockwave therapy for treating patellar tendinopathy: a randomized controlled trial. *Knee Surgery, Sports Traumatology, Arthroscopy*.

[26] Król P., Franek A., Durmała J. (2015). Focused and Radial Shock Wave Therapy in the Treatment of Tennis Elbow: A Pilot Randomised Controlled Study. *Journal of Human Kinetics*.

[27] Markus Dietmar S., Frank H., Christian Dominik P., Melanie A., SJAOB J. (2009). High- versus low-energy extracorporeal shock wave therapy of rotator cuff tendinopathy: a prospective, randomised, controlled study. *Orthopaedica Belgica*.

[28] Gerdesmeyer L., Wagenpfeil S., Haake M. (2003). Extracorporeal Shock Wave Therapy for the Treatment of Chronic Calcifying Tendonitis of the Rotator Cuff. *JAMA*.

[29] Speed C. A., Richards C., Nichols D. (2002). Extracorporeal shock-wave therapy for tendonitis of the rotator cuff: A double-blind, randomised, controlled trial. *The Journal of Bone and Joint Surgery. British volume*.

[30] Moya D., Ramón S., Schaden W., Wang C.-J., Guiloff L., Cheng J.-H. (2018). The role of extracorporeal shockwave treatment in musculoskeletal disorders. *The Journal of Bone and Joint Surgery*.

[31] BrañEs J., Contreras H. R., Cabello P., Antonic V., Guiloff L. J., Brañes M. (2016). Shoulder Rotator Cuff Responses to Extracorporeal Shockwave Therapy: Morphological and Immunohistochemical Analysis. *Shoulder & Elbow*.

[32] Tei K., Matsumoto T., Mifune Y. (2008). Administrations of peripheral blood CD34-positive cells contribute to medial collateral ligament healing via vasculogenesis. *Stem cells*.

[33] Feichtinger X., Monforte X., Keibl C. (2019). Substantial biomechanical improvement by extracorporeal shockwave therapy after surgical repair of rodent chronic rotator cuff tears. *The American journal of sports medicine*.

[34] Kenmoku T., Iwakura N., Ochiai N. (2020). Influence of different energy patterns on efficacy of radial shock wave therapy. *Journal of Orthopaedic Science*.

[35] Park K. D., Lee W. Y., Park M. H., Ahn J. K., Park Y. (2018). High- versus low-energy extracorporeal shock-wave therapy for myofascial pain syndrome of upper trapezius: a prospective randomized single blinded pilot study. *Medicine*.

[36] Mattacola C. G., Stiller J., Uhl T. L. (2005). Outcomes Measurement of Upper Extremity Function. *Athletic Therapy Today*.

[37] Patel V. R., Singh D., Calvert P. T., Bayley J. I. L. (1999). Arthroscopic subacromial decompression: Results and factors affecting outcome. *Journal of Shoulder and Elbow Surgery*.

[38] Miniaci A., Salonen D. (1997). ROTATOR CUFF EVALUATION: IMAGING AND DIAGNOSIS. *Orthopedic Clinics of North America*.

[39] Kjellin I., Ho C. P., Cervilla V. (1991). Alterations in the supraspinatus tendon at MR imaging: correlation with histopathologic findings in cadavers. *Radiology*.

[40] Seo K.-H., Lee J.-Y., Yoon K. (2018). Long-term outcome of low-energy extracorporeal shockwave therapy on gluteal tendinopathy documented by magnetic resonance imaging. *PLOS ONE*.

